# Research on non-destructive and rapid detection technology of foxtail millet moisture content based on capacitance method and Logistic-SSA-ELM modelling

**DOI:** 10.3389/fpls.2024.1354290

**Published:** 2024-05-30

**Authors:** Zhichao Qiu, Gangao Li, Zongbao Huang, Xiuhan He, Zilin Zhang, Zhiwei Li, Huiling Du

**Affiliations:** ^1^ College of Agricultural Engineering, Shanxi Agricultural University, Jinzhong, China; ^2^ College of Information Science and Engineering, Shanxi Agricultural University, Jinzhong, China; ^3^ Department of Basic Sciences, Shanxi Agricultural University, Jinzhong, China

**Keywords:** foxtail millet, moisture content, capacitance method, sensor, algorithm, modelling

## Abstract

Moisture content testing of agricultural products is critical for quality control, processing efficiency and storage management. Testing foxtail millet moisture content ensures stable foxtail millet quality and helps farmers determine the best time to harvest. A differential capacitance moisture content detection device was designed based on STM32 and PCAP01 capacitance digital converter chip. The capacitance method combined with the back-propagation(BP) algorithm and the extreme learning machine(ELM) algorithm was chosen to construct an analytical model for foxtail millet moisture content, temperature, and volume duty cycle. This work performs capacitance measurements on foxtail millet with different moisture contents, temperatures, and proportions of the measured substance occupying the detection area (that is, the volumetric duty cycle). On this foundation, the sparrow search algorithm (SSA) is used to optimize the BP and ELM models. However, SSA may encounter problems such as falling into local optimization solutions due to the reduction of population diversity in the late iterations. As a consequence, Logistic algorithm is introduced to optimize SSA, making it more appropriate for solving specific problems. Upon comparative analysis, the model predicted using the Logistic-SSA-ELM algorithm was more accurate. The results indicate that the predicted values of prediction set coefficient of determination (RP), prediction set root mean square error (RMSEP) and prediction set ratio performance deviation (RPDP) were 0.7016, 3.7150 and 1.4035, respectively. This algorithm has excellent prediction performance and can be used as a model for detection of foxtail millet moisture content. In view of the important role of foxtail millet moisture content detection in acquisition and storage, it is particularly important to study a nondestructive and fast online real-time detection method. The designed capacitive sensor with differential structure has well stabilization and high accuracy, which can be further studied in depth and gradually move towards the general trend of agricultural development of smart agriculture and precision agriculture.

## 1 Introduction

Foxtail millet is a major food crop in China, and how much water it contains after harvest is related to various aspects such as yield issues, sales price and storage with processing, while at the same time, it is also an essential indicator of the quality of the foxtail millet. Excessive moisture content of foxtail millet can easily lead to mold, germination, deterioration and other problems, so that it loses its commodity value, reduces the nutritional value, and even causes hidden food safety hazards; while too low a moisture content will interfere with the nature of the processing and storage, increase energy consumption, and negatively affect the efficiency of production (Qian et al., 2018; Zeyu et al., 2021). Therefore, the determination of foxtail millet moisture content is an integral part of grain science and technology and processing production. To this end, this paper designs a differential capacitance sensor which can detect moisture content online in real time. This can be used to infer the foxtail millet quality within a small area based on the online real-time detection results and further develop towards modernized precision agriculture. In this regard, it is of great significance to conduct accurate online real-time measurement of foxtail millet moisture content.

At present, the research of online moisture content detection system in foreign countries is relatively mature, but the domestic research on this aspect started late, and the application is few. However, after many years of experimental analysis, domestic scholars have also achieved some obvious results in moisture content detection. Liu Jin et al. designed a portable grain moisture content detection device based on a microstrip microwave sensor. The results show that when microwave attenuation, phase shift and temperature are selected as inputs to the Random Forest Algorithm model, the prediction results show the best accuracy and stability, with a maximum average absolute error of 0.55 per cent and a maximum standard deviation of 0.41 per cent. The device can be well applied to the moisture content detection of three kinds of grains: rice, soybean and wheat. The designed portable grain moisture content detection device is small in size, light in weight, fast and accurate in detection results, and provides important reference significance for real-time measurement of agricultural products and the development and application of intelligent agricultural equipment (Jin et al., 2023). Zhang Y et al. introduced a new approach based on near-infrared (NIR) hyperspectral imaging for the detection of moisture content in maize seeds and investigated the extraction of the centre of mass region using averaged spectra. The evidence suggests that the PLSR model built by extracting the average spectrum from the center-of-mass region performs well and has a high potential (Zhang and Guo, 2020). Based on the principle of dielectric properties of wheat, An Xiaofei et al. designed an on-line moisture detection device for combine harvester, which realized fast and stable on-line detection of wheat moisture content under the operating conditions of combine harvester. The test results showed that the online detection error of moisture content was within 3% under static conditions. Under the dynamic change conditions in the field, a moisture detection model based on dielectric constant and temperature factor was established, and the correlation coefficient between measured and detected values reached 0.92, and the online detection error was less than 5 per cent. The method of dynamic continuous sampling and static intermittent measurement significantly improves the accuracy of on-line detection of moisture content, and provides a rapid measurement means for realizing accurate wheat production (Xiaofei et al., 2022). Wang Xiao et al. designed and processed a microstrip patch antenna to achieve non-contact, real-time and high-precision water content detection using rice as the detection object. Using both contact and non-contact detection methods, the relationships among rice water content, bulk density, detection height and resonance frequency, return loss and phase were investigated. The detection sensitivity of the proposed microstrip antenna is characterised by resonance frequency, return loss, and phase as 600 kHz/%, 0.149 dB/%, and 1100 kHz/%, respectively, and the minimum average relative error of detection is 0.026%, 0.083%, and 0.028%, respectively. The results demonstrate that the microstrip antenna has special advantages in grain moisture content detection, which provides an important reference for real-time moisture content detection during grain storage and transport (Xiao et al., 2021). By determining the hyperspectral reflectance and water content of summer maize leaves, Zheng Zhikang et al. constructed spectral indices in any two bands using the original and converted spectra and analysed the relationship between spectral indices and leaf water content. The results showed that the spectral reflectance in the short-wave infrared band decreased with the increase of leaf water content, and the constituent bands of the optimal spectral indices were mainly located in the short-wave infrared band, among which the ratio spectral indices based on the first-order derivative spectra (R_1 563_/R_1 406_) and the normalised spectral indices [(R_1 563_ - R_1 406_)/(R_1 563_ + R_1 406_)] had the best correlation with leaf water content, with correlation coefficients of 0.83 in absolute value. The multifactor regression model was simulated better than the single-factor regression model, and the sparrow search-based random forest regression model had the highest accuracy, with a validation set coefficient of determination (R^2^) of 0.78, and root-mean-square error (RMSE) and relative error (RE) of 1.14 per cent and 1.09 per cent, respectively. In this study, a remote sensing estimation model was established by analysing the relationship between maize leaf water content and hyperspectral reflectance to provide a basis for water management of summer maize production in Guanzhong region (Zhikang et al., 2023).

The significance of using physical modelling i.e. capacitive sensors combined with algorithms to predict moisture content in this study is to achieve real-time monitoring and prediction of moisture content, which can provide real-time data support in multiple fields. It can help to improve the efficiency of resource utilization, reduce costs, and achieve the goal of sustainable development, thus providing important information and application value in a variety of fields. The common methods of measuring moisture content mainly include direct drying oven method, resistance method, ray method, microwave method (Lin et al., 2022), near infrared method (Chen et al., 2017), capacitance method (Fridh et al., 2018; Deng et al., 2020; Oommen and Philip, 2023), etc. Drying oven method which measures moisture content has accurate measurement results, but this method is time-consuming and not easy to realize on-line real-time detection, normally the measurement results of this drying method are used as the standard results (jing et al., 2018). Resistance method of measuring moisture content is inexpensive and has a rapid test speed, but it is limited by the influence of material distribution, which leads to low signal strength and low accuracy (Shibiao et al., 2019). Ray method of measuring moisture content detects with fast speed, wide range and excellent penetrability, which can quickly carry out non-destructive testing of the moisture content of the measured substance. However, there are radiation hazards in the ray, and the equipment is costly, which is not favourable to the agricultural testing environment (Yitong et al., 2021). Microwave method of measuring water content has low energy consumption, high testing speed and superior anti-interference ability. Nevertheless, the lower limit of detection is insufficiently low, which may easily cause standing wave interference. Meanwhile, the measured value is associated with the composition of materials, and different varieties need to be calibrated individually (Chenyu, 2023). Near infrared method to measure the moisture content analytic rate is fast, no damage to the test sample, but the detection accuracy is affected by the test sample particle size, density and other factors (Leblon et al., 2013).

Considering the shortcomings of the above methods, this paper applied the capacitance method and designed a differential capacitance sensor to measure the moisture content. The capacitance method for measuring water content provides relatively low cost, with a fast response time and comparatively simple structure, which can satisfy the accuracy requirements. The principle is according to varying dielectric constants of foxtail millet with different moisture content, there will be a difference in the capacitance value when passing through the two sides of the pole plate (Yin, 2018; Nath and Ramanathan, 2020; Hao et al., 2021; Lev et al., 2021; Danyang et al., 2022; Shekhar and Prasad, 2023). Analyse the change of capacitance value when the moisture content of foxtail millet, ambient temperature, and volumetric duty cycle are varied. And then, a relevant mathematical model is established, and the model is used as a benchmark in order to calculate the moisture content of the foxtail millet.

**Figure 1:**
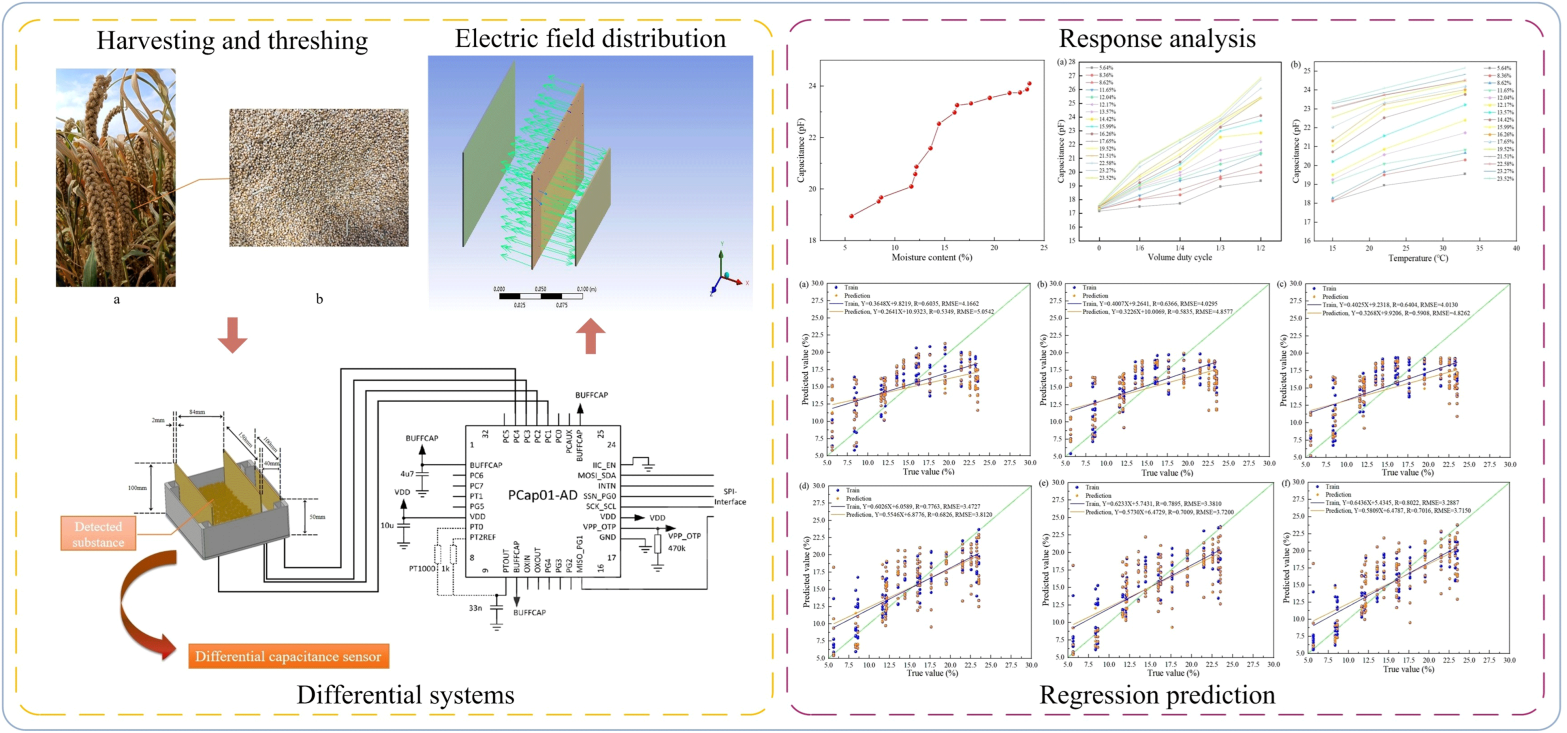
Graphical abstract.

A graphical abstract of this paper is shown in **Figure 1** below:

## 2 Material and methods

### 2.1 Experimental materials

**Figure 2:**
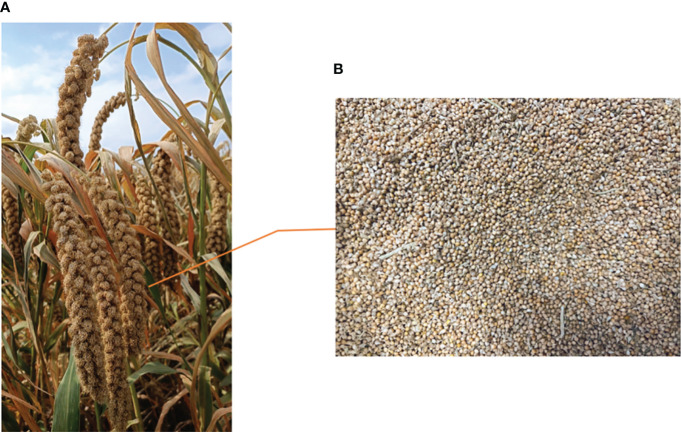
The state of foxtail millet before and after threshing. Figure **(A)** shows the state of the foxtail millet ears. Figure **(B)** shows the state of the foxtail millet ears after threshing.

The test site was Taigu District, Jinzhong City, Shanxi Province, which has a warm temperate continental climate. Spring has a higher temperature than fall, while summer is warm, hot and rainy, as well as long and cold in winter. In this experiment, Zhangza foxtail millet planted in Taigu District, Jinzhong City, Shanxi Province was used as the sample, which was collected at the beginning of October, 2022. Since the experiment tested the moisture content of the cereal granules, the earhead was required to be threshed as shown in **Figure 2**. Wherein, **Figure 2A** shows the state of the foxtail millet ears, and **Figure 2B** shows the state of the foxtail millet ears after threshing. The harvested foxtail millets were randomly divided into 48 samples of 720g each. Simultaneously, each sample was placed in a plastic self-sealing bag (size 240 mm × 350 mm) so as to prevent the evaporation of water. Thereafter, they were stored at room temperature of 22°C.

A total of 16 different gradients of moisture content were formulated for this study and each of them was divided into three samples for testing experiments, summarizing a total of 48 foxtail millet samples. The initial moisture content of the harvested foxtail millets was measured to be 14.42% using a rapid moisture meter (Model HM-101X, Shanghai Hegong Scientific Instrument Co., Ltd., precision 0.001g). Weighing was done using a balance (Model ACS-30, Shanghai Kaishi Electronics Co., Ltd., 10g divisional value) with 720g for the each portion. In order to prepare the samples with different moisture contents of the gradient, the weighed foxtail millet samples were taken out first. After that, the samples that are higher than the initial moisture content were obtained by spraying deionized water, and the samples that are lower than the initial moisture content were obtained by placing them in an electric thermostatic blower drying oven (GZX-GF101-2-BS-II/H type, Shanghai Yuejin Medical Equipment Co., Ltd., max. temperature 300°C) in various times, set the temperature to 105°C. And cool them down to room temperature. Using the rapid moisture meter again, the remaining 15 kinds of moisture content were measured as 5.64%, 8.36%, 8.62%, 11.65%, 12.04%, 12.17%, 13.57%, 15.99%, 16.26%, 17.65%, 19.52%, 21.51%, 22.58%, 23.27%, and 23.52%, respectively. Ultimately, a sum of 16 kinds of foxtail millet samples with different moisture contents were acquired and numbered. For ensuring uniform water absorption in each sample, the prepared samples were put into plastic self-sealing bags. They were set in a room temperature environment at 22°C for 1 to 2 days. During this period, the samples were removed 3 to 4 times a day, stirred thoroughly, then poured back into the bag and sealed well again. This ensures that the moisture in each sample is individually distributed evenly.

### 2.2 Differential capacitance sensor detection principle

In this study, a capacitive sensor with differential structure is designed to be constructed on the basis of a capacitive sensor with parallel plate structure. Differencing is the method of subtracting the two adjacent values in a series of output data to obtain the amount of change in the two adjacent values. In the process of data analysis, only the results after differencing are analysed. This means that only changes between successive data are analysed, ignoring trends or seasonality formed through the accumulation of the data itself. Thus, the role of differencing is to mitigate irregular fluctuations between the data and make their fluctuation curves smoother. It is also capable of minimizing the negative impact of external disturbances, such as environmental factors, on the measured capacitance value. The differential handling of the data yields an increment of the data rather than the data itself, and generally the data will be more stable after first-order differencing, so the differenced data is used for analysis (Heming et al., 2019).

Since water and foxtail millet have distinct dielectric properties, variations in the capacitance values detected by the sensor arise when foxtail millets with different moisture contents fill the detection area.

While the foxtail millets fulfil the analyzed moisture content condition, the sensor output capacitance *C* is:


(1) $$C=\frac{S\varepsilon_{0}\varepsilon_{r}}{d}$$

Where, *S* is the relative area of the pole plate in m^2^; $\varepsilon_{0}$ is the vacuum dielectric constant, which is specified in the International System of Units as $\varepsilon_{0}$ =8.854187818×10^-12^ F/m; $\varepsilon_{r}$ is the relative permittivity of the foxtail millets in the detection area; *d* is the pole plate spacing in m.

The inter-polar plate medium is composed of air and foxtail millets, while the foxtail millets contain varying amounts of water, so that the total volume *V* can be expressed as:


(2) $$V=V_{cereal}+V_{water}+V_{air}$$

(3) $$\varepsilon_{r}=\frac{V_{cereal}}{V}\varepsilon_{cereal}+\frac{V_{water}}{V}\varepsilon_{water}+\frac{V_{air}}{V}\varepsilon_{air}$$

Where, $V_{cereal}$ is the volume occupied with dry foxtail millets in the detection area in m^3^; $V_{water}$ is the volume occupied with moisture in the detection area in m^3^; $V_{air}$ is the volume occupied with air in the detection area in m^3^; $\varepsilon_{cereal}$ is the relative permittivity of dry foxtail millets; $\varepsilon_{water}$ is the relative permittivity of moisture; $\varepsilon_{air}$ is the relative permittivity of air. Substituting Equation 3 into Equation 1 to get Equation 4 which can be written as:


(4) $$C=\frac{S\varepsilon_{0}}{d}(\frac{V_{cereal}}{V}\varepsilon_{cereal}+\frac{V_{water}}{V}\varepsilon_{water}+\frac{V_{air}}{V}\varepsilon_{air})$$

(5) $$e=\frac{V_{air}}{V}$$

Here, e is the foxtail millet pore ratio in the detection area. According to the formula for the calculation of moisture content, it is known that the moisture content W of the foxtail millet can be written as:


(6) $$W=\frac{\rho_{water}V_{water}}{\rho_{cereal}V_{cereal}+\rho_{water}V_{water}}\times 100\%$$

Where, $\rho_{cereal}$ is the dry foxtail millet density in kg/m^3^ and $\rho_{water}$ is the moisture density in kg/m^3^.

Once the capacitive sensor dimensional structure is specified, S , e , and d are determined. Furthermore, $\rho_{cereal}$ , $\rho_{water}$ , $\varepsilon_{0}$ , $\varepsilon_{cereal}$ , $\varepsilon_{water}$ , and $\varepsilon_{air}$ are determined by the substance itself and are known values. In this study, $\rho_{cereal}$ =600kg/m^3^, $\rho_{water}$ =10³kg/m³, $\varepsilon_{cereal}$ =3, $\varepsilon_{water}$ =81, $\varepsilon_{air}$ =1.00053. Thus *A*, *B*, *D*, and *F* can be expressed as:


(7) $$A=\frac{S\varepsilon_{0}\rho_{water}(1-e)(\varepsilon_{cereal}-\varepsilon_{water})}{d\rho_{cereal}}$$

(8) $$B=\frac{\rho_{water}}{\rho_{cereal}}$$

(9) $$D=\frac{S\varepsilon_{0}}{d}(1-e)\varepsilon_{water}$$

(10) $$F=\frac{S\varepsilon_{0}}{d}e\varepsilon_{air}$$

According to Equations 4–10, the capacitance *C* can be expressed as the following equation:


(11) $$C=A(\frac{W}{1-W}+B)+D+F$$

In the formula, *A*, *B*, *D* and *F* are all structural constants of the sensor.

From Equation 11, it can be observed that the value of moisture content of the foxtail millet can be derived from the value of capacitance *C*.

During the capacitive sensor functioning, the relative dielectric constants $\varepsilon_{cereal}$ , $\varepsilon_{water}$ , and $\varepsilon_{air}$ are associated with the temperature (Jinwu et al., 2021). Therefore, for this affecting factor, it should be considered.

At room temperature, the relative dielectric constant of foxtail millets is 2.5 to 4.5, while the relative dielectric constant of water is 81. Obviously, when the moisture content of the foxtail varies, there will be a consequent change in its relative dielectric constant, which will affect the capacitance value. It can be seen that both are positively proportional to each other. When foxtail millets with different moisture content are placed in the detection area, $\varepsilon_{r}$ is different, which in turn affects the capacitance value of the output in the detection area, and the moisture content of the foxtail millets can be estimated according to the corresponding mathematical model.

### 2.3 System design

An electrode line is drawn from each of the two pole plates on each side of the detection area, and from each of the other two pole plates that form a differential structure with it, to connect to the PCAP01 capacitive-digital converter chip. The chip covers a measurement range from a few fF to several hundred nF with high measurement accuracy, low power consumption and extremely fast measurement speed. As a result, it has a wide range of applications.

(1) Differential capacitance sensor design.

**Figure 3:**
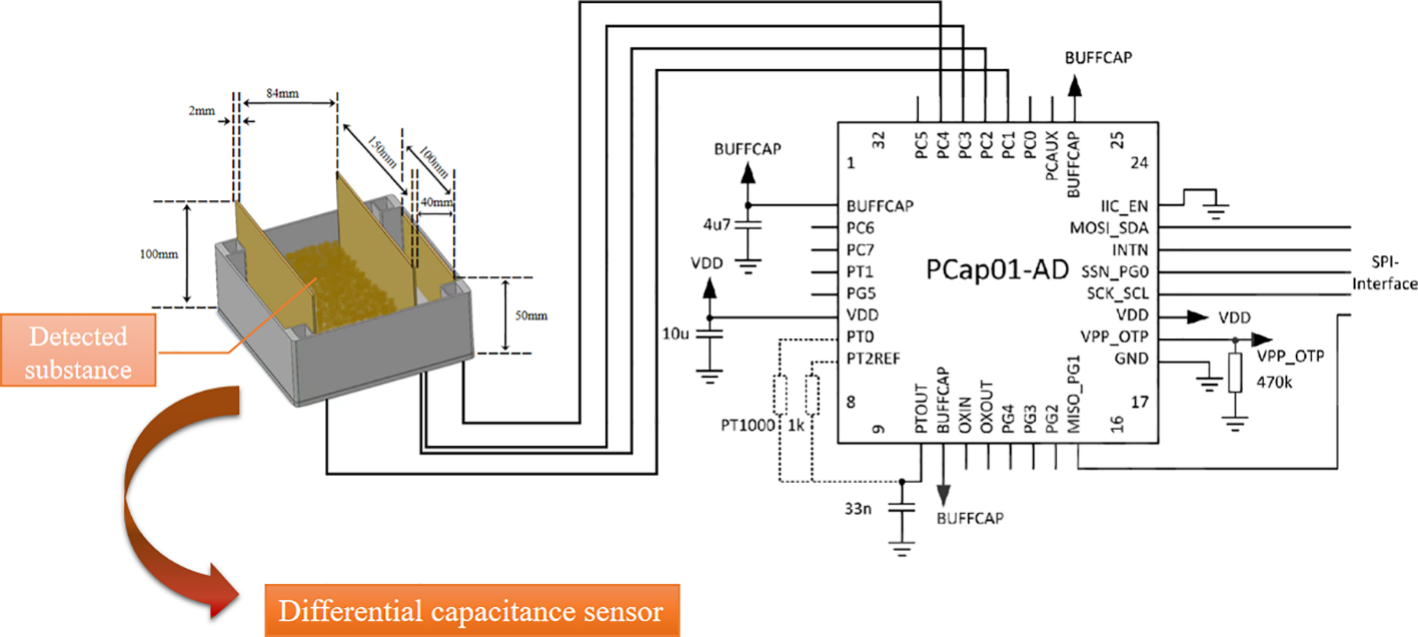
Schematic diagram of differential capacitance sensor.

The designed differential capacitive sensor is composed of two pieces of brass plates 150mm long and 100mm wide as well as two pieces of brass plates 100mm long and 50mm wide, both 2mm thick. This structure consists of two pairs of pole plates with a total of two capacitance detection areas. One way is the detection capacitance and the other way is the reference capacitance, that is to say, it constitutes a differential structure. The designed differential structure capacitance sensor can attenuate the interference and enhance the detection accuracy of the sensor with high sensitivity and fine stability. The principle of the capacitance sensor as shown in **Figure 3**.

(2) Distribution of the sensor electric field.

Edge effects in electric fields refer to the phenomenon of the existence of charges or electric fields at the edges or margins of electrodes (Wei et al., 2022). At the edges of the electrodes, there is an increase or decrease in charge density due to uneven charge distribution on the electrode surface. This affects the potential and electric field distribution throughout the electrode. Again, changes in charge density can make the electric field stronger or weaker at the edges, causing the electric field distribution to appear more sophisticated at the edges, which in turn affects the performance of the electrodes and the measurement results.

**Figure 4:**
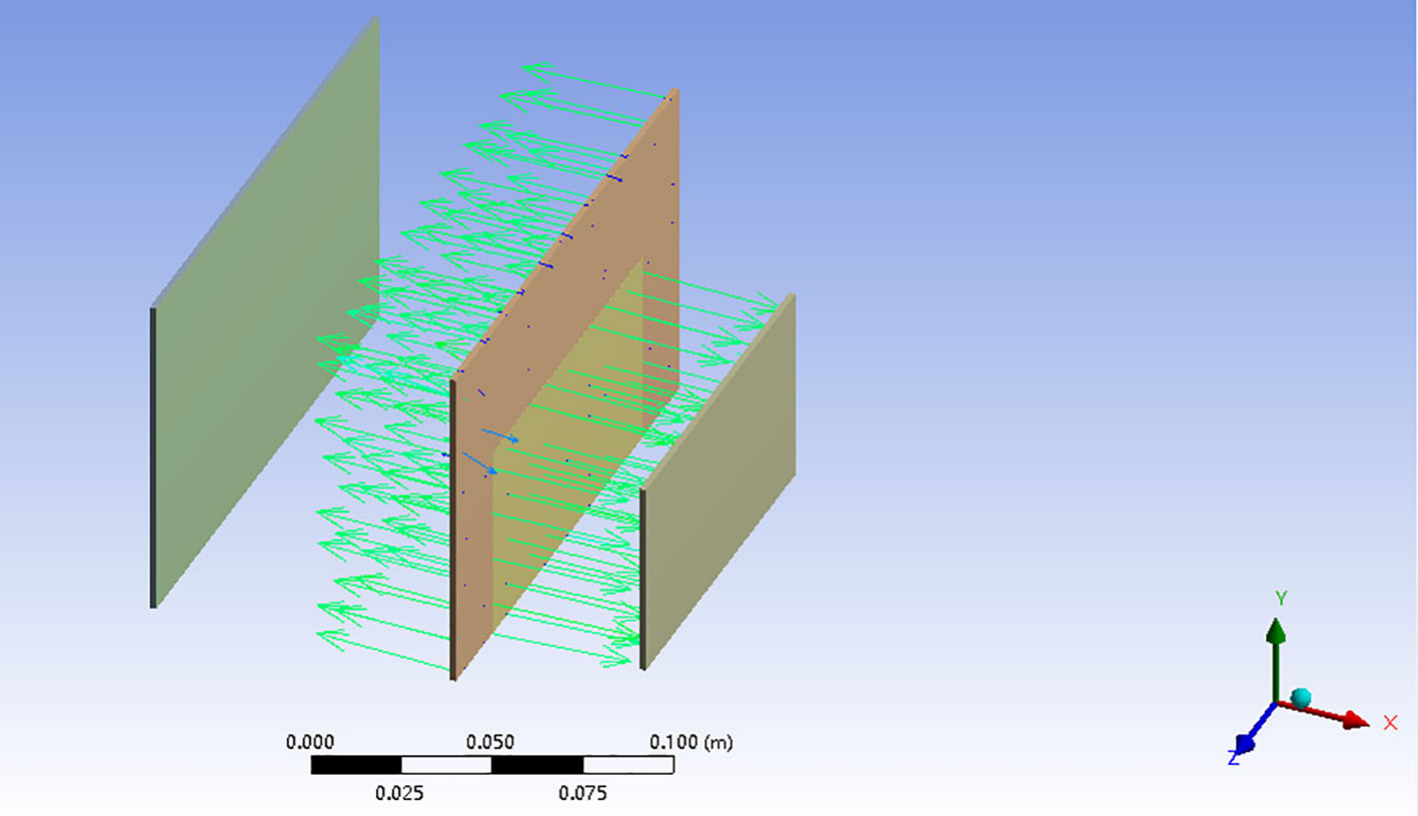
Electric field distribution of differential capacitive sensor.

In view of this above, the electric field simulation of the designed differential structure capacitance sensor is carried out using ANSYS software. The primary steps of the simulation are as follows: utilize SolidWorks to establish the model, create a new electric field analysis in Workbench, and import the model; add copper material in Engineering Data Sources; enter the model module, add environment variables; build local coordinate system of the new model, modify the model’s coordinate system and the material; carry out mesh delineation, and its Element Size is 5mm; apply the current load, Magnitude is set to 4uA; then install the air-domain magnetic flux parallel boundary conditions; set the current density to view the result model, and the electric field strength of the whole result, and lastly, perform the solution. The result is displayed in **Figure 4**. From the solution results, it can be seen that the differential capacitive sensor electric field distribution is fairly uniform, while getting uniform electric field distribution, the differential structure still cripples the influence of external interference, which can broaden the application field.

### 2.4 Data processing and sample set division

This study focuses on the effect of foxtail millet moisture content, ambient temperature, and volumetric duty cycle on capacitance. The above proportioned foxtail millets with moisture content ranging from 5.64% to 23.52% were subjected to ambient temperatures of 15°C, 22°C and 33°C, with the volume duty cycle set to 0, 1/6, 1/4, 1/3 and 1/2, respectively. The capacitance values collected for each sample at three temperatures and five volume duty cycles, separately, were detected using the designed sensor. Parallel experiments were conducted in each group and repeated three times, which not only prevented the generation of chance errors, but also observed the stability of the sensors during detection. Observe the acquired data so as to search for some abnormal data owing to the test operation and instrumentation, and re-test them after eliminating them. Eventually, 720 sets of capacitance data were obtained. Wherein, the capacitance value data for performing model training is obtained by the following steps: firstly, the output capacitance of foxtail millets with different moisture content in the detection area of the larger size pole plate is recorded. Secondly, the output capacitance of air detected by the smaller size pole plate is recorded. Finally, the two simultaneous output capacitance signals are differenced to obtain the training data for the final model. Generally, when performing model construction, the dataset is divided into training set, validation set and test set. However, in this study, the dataset is directly divided into training and testing sets due to the fact that too much segmentation in a smaller dataset results in a smaller training set, which may lead to overfitting (Ashtiani et al., 2021). In order to provide sufficient training data, this study uses a test set to evaluate the performance of the model. Randomly selected 3/4 of the collected data as the training set for model establishment, and the other 1/4 of the data as the prediction set. The results of their division are shown in **Table 1**.

**Table 1:** Statistics of foxtail millet moisture content data set.

Data set	NS^a^	XV^b^(%)	NV^c^(%)	AV^d^(%)	SD^e^(%)
**Training set**	540	23.52	5.64	15.2467	5.5087
**Prediction set**	180	23.52	5.64	15.95247	5.1262

NS^a^, Number of samples; XV^b^, Maximum value; NV^c^, Minimum value; AV^d^, Average value; SD^e^, Standard deviation.

### 2.5 Modeling methodology and evaluation indicators

The Extreme Learning Machine (ELM) algorithm and the Backpropagation algorithm (BP) are both common machine learning algorithms that are capable of handling complex nonlinear relationships. Therefore, they are able to show excellent performance in many practical problems. At the same time, it is flexible and scalable, and can be adapted to a variety of different problems and tasks. And the performance and complexity of the model can be improved by adding hidden layers or adjusting the network structure. In some cases, both algorithms have superior generalisation capabilities and are able to accurately predict or classify unseen data. Therefore, they are a vital choice of algorithms in the field of machine learning. In this study, these two algorithms are selected for modelling according to the characteristics of the specific problem and dataset, combined with the actual application scenarios and requirements, and weighed and compared to finally select the optimal model.

Extreme Learning Machine (ELM) is a rapid, simplistic and efficacious artificial neural network algorithm. The ELM initializes the connection weights between the input and hidden layers in a random manner, and then maps the input signals to the hidden layers using a high-dimensional nonlinear function. After the mapping is complete, the ELM quickly learns the weights of the output layer by least squares or regularization methods to approximate the objective function (Li and Wu, 2022; Qiaoyun et al., 2023). Compared with traditional neural networks, ELM does not require iterative weight adjustment, has fast training speed and well generalization ability.

Back Propagation (BP) is a popular algorithm which is used to train neural networks. The BP algorithm is based on the gradient descent method, where the weights and biases of the neural network are updated through continuous iterations thereby minimizing the loss function. The BP algorithm first calculates the predicted output of the network through forward propagation, then calculates the error between the predicted output and the actual output through back propagation and passes that error back to the network. The gradient of each layer is calculated in accordance with the chain rule and the weights are updated (Chen et al., 2022b; Li et al., 2022; Zhijun et al., 2022; Lihua et al., 2023). This process is iterated until the loss function is minimized.

Sparrow Search Algorithm (SSA) is a population optimization algorithm. Based on observing the local optimal solution of the target problem, the SSA algorithm iteratively searches for the global optimal solution, which has the characteristics of global exploration and local optimization (Dong et al., 2022; Yan et al., 2022; Yue et al., 2023). Through the global search strategy and the ability to regenerate the initial solution, the sparrow search algorithm can help ELM and BP algorithms to jump out of the local optimal solution and discover a better combination of weights. Moreover, the sparrow search algorithm can also optimize the current solution through local search, and gradually improve the accuracy of the weight (Gao et al., 2022). Taken together, the sparrow search algorithm is more flexible in the global and local optimization process and has the advantages of fewer iterations, faster convergence speed and higher search efficiency.

During the search for food in sparrow populations, populations are laid out in synergy in the form of predators, followers, and early warners. The *n*×*d* dimensional vector population consisting of *n* sparrows is represented by a matrix, which can be expressed as follows in Equation 12:


(12) $$X=\left(\begin{array}{cccc} X11 & X12 & \ldots & X1d\\ X21 & X22 & \cdots & X2d\\ ⫶ & ⫶ & ⫶ & ⫶\\ Xn1 & Xn1 & \cdots & Xnd \end{array}\right)$$

Where, *X_ij_* is the *j*th dimensional position of the *i*th sparrow; *n* is the number of individual sparrows in this sparrow population; *d* is the dimension of the variable space of the objective function. The fitness of the sparrow population can be expressed as follows in Equation 13:


(13) $$Fx=\left(\begin{array}{c} f\left(\left(\begin{array}{cccc} X11 & X12 & \cdots & X1d \end{array}\right)\right)\\ f\left(\left(\begin{array}{cccc} X21 & X22 & \cdots & X2d \end{array}\right)\right)\\ ⫶\\ f\left(\left(\begin{array}{cccc} Xn1 & Xn2 & \cdots & Xnd \end{array}\right)\right) \end{array}\right)$$

Where, *f* is the individual fitness of the sparrow.

The SSA algorithm in the merit seeking process, the discoverer with higher fitness will have priority in acquiring food during the iterative search process. Since discoverers provide foraging search direction for the entire population, discoverers have a larger search range than joiners (Tang et al., 2023). In the iterative process, the finder position update formula is as follows in Equation 14:


(14) $$X_{Fi,j}^{t+1}=\{\begin{array}{c} X_{Fi,j}^{t}\cdot \exp\left(-\frac{i}{\alpha T}\right)\;R<ST\\ X_{Fi,j}^{t}+Q\;R\geq ST \end{array}$$

Where, *t* is the current number of iterations; *T* is the maximum number of iterations; *X_Fi,j_* is the position of the *i*th sparrow in the *j-*th dimension. *α*∈ (0, 1] are uniform random numbers. *R*∈[0, 1] and *ST*∈[0.5, 1] are the early warning values and safety values, respectively. *Q* is a random variable obeying a normal distribution. When *R*<ST, no natural enemies are found in the vicinity of the population and the foraging environment is safer. At this point the discoverer can conduct an extensive search. When *R*≥ST, part of the sparrows in the population have detected the predator and started to alert the other sparrows in the population. The population tunes into an anti-predator mode and needs to seek a safe area as soon as possible (Chen et al., 2022a).

For joiners, whose behavioural characteristics are influenced by the discoverer, the location update formula as Equation 15:


(15) $$X_{Ji,j}^{t+1}=\{\begin{array}{c} Q\cdot \exp\left(-\frac{X_{L}^{t}-X_{Ji,j}^{t}}{i^{2}}\right)\;i>0.5n\\ X_{P}^{t+1}+\left|X_{Ji,j}^{t}-X_{P}^{t+1}\right|\cdot L\cdot A^{+}\;i\leq 0.5n \end{array}$$

Where, *X_P_* is the current optimal position in which the discoverer is located; *X_L_* is the current global worst position; *L* is a matrix of dimension 1*×d* and all elements are 1; *A* is a matrix of dimension 1*×d* and each element of which is randomly 1 or -1, and *A^+^=A^T^* (*AA^T^*). While *i>*0.5*n*, the *i*th joiner is less acclimatized and does not receive food, is in a very starved state, which requires flying to other regions in order to replenish its energy. While *i ≤* 0.5*n*, the *i*th joiner will forage randomly in the vicinity of *X_P_*.

Throughout the population, some of the sparrows serve as early warning scouts, responsible for spreading warning signals to the entire population, and thereby leading the population to a new safe area. Sparrows accounting for 10% ~20% of the total population are randomly selected in each generation of the population to perform the early warning function, and their location update formulas are as follows in Equation 16:


(16) $$X_{Di,j}^{t+1}=\{\begin{array}{c} X_{B}^{t}+\beta \cdot \left|X_{Di,j}^{t}-X_{B}^{t}\right|\;f_{i}>f_{g}\\ X_{Di,j}^{t}+K\cdot \left(\frac{\left|X_{Di,j}^{t}-X_{L}^{t}\right|}{\left(f_{i}-f_{w}\right)+\varepsilon }\right)\;f_{i}\leq f_{g} \end{array}$$

Where, *X_B_* is the current global optimal position; *f_i_* is the fitness of the current sparrow individual; *f_g_* as well as *f_w_* are the current global optimal and worst sparrow individual fitnesses, respectively. *β* is a step control parameter that obeys a normally distributed random number with variance 1 and mean 0. *K*∈[-1, 1] is a random number, an infinitesimal constant, mainly to avoid zeros in the denominator of the fraction. While *f_i_>f_g_*, the sparrow is at the edge of the population and is vulnerable to predators. While *f_i_* ≤*f_g_*, the sparrow in the middle of the population realizes the danger, which requires approaching other sparrows in the population so as to reduce the probability of predation (Xue and Shen, 2020).

Sparrow search algorithm (SSA) may encounter some problems in solving optimization problems such as getting stuck in the local optimal solution, failing to find the global optimal solution, convergence may be slower, requiring more iterations to reach the optimal solution, and the performance is highly dependent on the selection of parameters like the generation of the initial solution and the scope of the search, and some other issues, which can be optimized by Logistic algorithms (Wanli et al., 2019; Dingjie et al., 2021; Xin et al., 2021). Logistic chaotic mapping is a typical representative of chaotic mapping, which is more extensively applied due to its simple mathematical form. Logistic chaotic mapping is used to particle swarm algorithm, which can optimize the initial population. The mathematical expression is as follows in Equation 17:


(17) $$Y_{n+1}=aY_{n}\left(1-Y_{n}\right)$$

Here, *Y_n_* ∈[0, 1] and *a*∈[0, 4] are the Logistic parameters.

As *a* gets closer to 4, the range of values of *Y* is more nearly evenly distributed over the entire [0, 1] region. When *a* is taken as 4, the system is in a completely chaotic state and the uniformity of the mapping distribution reaches an extreme value. That is, with the initial condition *Y*_0_, the sequence generated by the Logistic mapping is non-periodic and non-convergent. Outside this range, the sequence must converge to a particular value. With the increase of *a*, the value of *Y* tends to be uniformly distributed in the interval [0, 1] (Andi et al., 2021). Applying Logistic chaotic mapping to SSA increases the homogeneity of the initial solution distribution, enhances the optimization efficiency and traversal uniformity, as well as improves the population search capability. In addition, it also overcomes the shortcomings of the swarm intelligence algorithm to a certain extent, such as the reduction of population diversity when approaching the optimal solution, the tendency to fall into the local optimum, and the reduction of search accuracy.

In summary, the Logistic algorithm can optimize the problems encountered by SSA in finding the global optimal solution, accelerating the convergence speed and optimizing the parameter selection, improving the optimization ability and efficiency of SSA so that it is more suitable for solving specific problems (Wang et al., 2021; Zhang et al., 2022).

In order to strengthen the accuracy and stability of the prediction model, Logistic algorithm is used to optimize Sparrow Search Algorithm (SSA), and then Back Propagation (BP) algorithm and Extreme Learning Machine (ELM) algorithm are optimized again individually. After that, the input and output layers are modeled and analysed. Using the test set correlation coefficient R as the model evaluation index, the inverse estimation model that can accurately predict foxtail millet moisture content was preferred after comparative analysis. Ambient temperature, volumetric duty cycle, and detected capacitance values were used as independent variables, and foxtail millet moisture content was used as the dependent variable to establish a prediction model. The evaluation metrics of the prediction model are correlation coefficient (R), root mean square error (RMSE) and relative percent deviation (RPD). The expressions are given in the following Equations 18–20. A larger correlation coefficient R of the prediction model indicates a higher correlation. The smaller the RMSE of the prediction model is, the better the prediction effect of the model is. When RPD< 1.4, the constructed model is regarded as unreliable. When 1.4< RPD< 2.0, the constructed model is regarded as relatively reliable. When RPD > 2.0, the constructed model is regarded as having high reliability and can be taken into account for model analysis (Tian et al., 2023). In this study, when using the ELM algorithm, the number of hidden layers was set to 100, the number of populations was set to 20, and the maximum number of iterations was set to 20. When using the BP algorithm, the number of nodes in the input layer was set to 3, the number of nodes in the output layer was set to 1, the number of nodes in the hidden layer was set to 5, the number of populations was set to 20, and the maximum number of iterations was set to 20, and the target error for the training of the neural network was 0.01.


(18) $$R=\frac{\sum_{i=1}^{N}\left(x_{i}-\bar{x})(y_{i}-\bar{y}\right)}{\sqrt{\sum_{i=1}^{N}{\left(x_{i}-\bar{x}\right)}^{2}}\sqrt{\sum_{i=1}^{N}{\left(y_{i}-\bar{y}\right)}^{2}}}$$

(19) $$RMSE=\sqrt{\frac{1}{N}\sum_{i=1}^{N}{\left(y_{i}-{\hat{y}}_{i}\right)}^{2}}$$

(20) $$RPD=\frac{1}{\sqrt{1-R^{2}}}$$

Where, *x_i_* is the moisture content data; $\bar{x}$ and $\bar{y}$ are the average values of the corresponding variables; *y_i_* is the actual value; $\hat{y_{i}}$ is the predicted value; *N* is the number of samples.

## 3 Results and analysis

### 3.1 Effect of moisture content on foxtail millet capacitance

**Figure 5:**
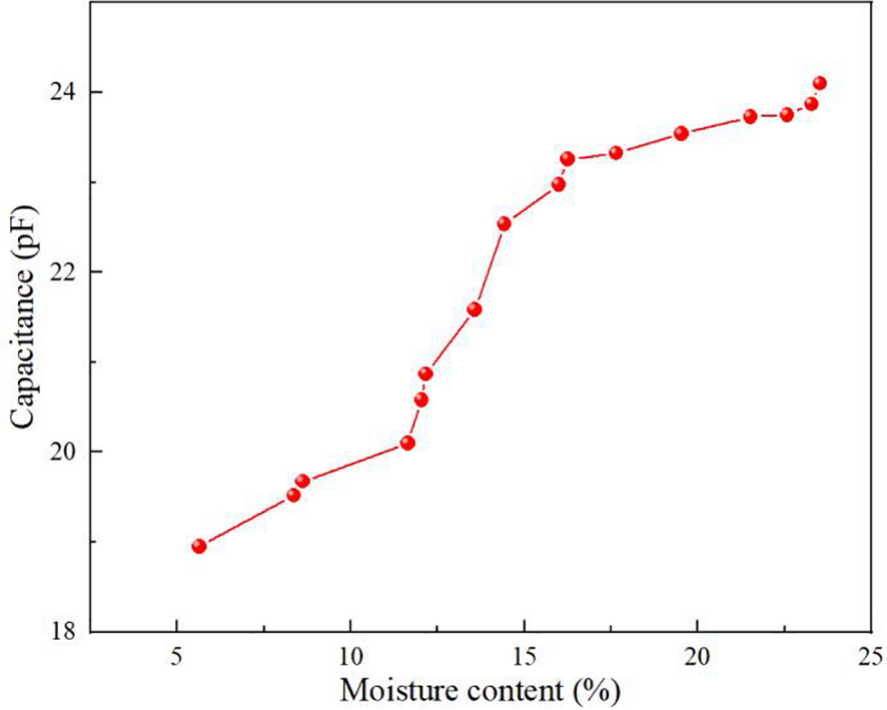
The relationship among moisture content and capacitance values.


The capacitance variation curves for foxtail millets with different moisture contents at the same temperature (22°C) and the same volume duty cycle (1/3) are shown in **Figure 5**. This is due to the fact that the capacitance of different samples is affected variously by the moisture content of the wet base, which is mainly manifested by the fact that the higher the moisture content of the wet base of the foxtail millet sample, the higher the capacitance. Foxtail millet moisture content refers primarily to the amount of internal free water. While the moisture content is low, the foxtail millets are mainly bound water inside, the intensity of cellular respiration is weak, the intracellular ionic movement is not active, and the effect of moisture on the capacitance is not significant. Along with the increase of moisture content, the free water content increases and eventually extends to the outside to form a multilayer molecular membrane, and the dipole moment then becomes larger. At the same time, cellular respiration is strengthened and internal ionic activity is enhanced, at which time the capacitance tends to increase (Pan et al., 2016; Chengjie et al., 2021; Guangyu et al., 2021; Xianglin et al., 2022). The specific relationship is shown in Equation 21:

(21) C=0.30744W+17.28085

Where *C* denotes the capacitance value and *W* denotes the foxtail millet moisture content.

### 3.2 Effect of temperature and volume duty cycle on foxtail millet capacitance

**Figure 6:**
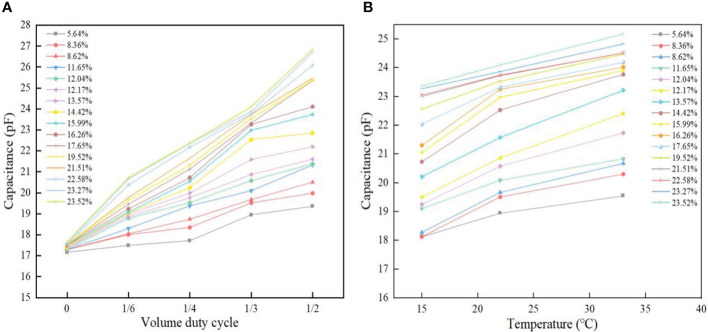
Effect of volume duty cycle and temperature on foxtail millet capacitance. Figure **(A)** demonstrates the volume duty cycle versus capacitance. Figure **(B)** demonstrates temperature versus capacitance.

**Figure 6** shows the curves on the effects of volumetric duty cycle (which can also be called volumetric concentration) and temperature for the foxtail millet capacitance.

It can be seen from **Figure 6A** that at a certain temperature (22°C) and constant moisture content, the foxtail millet capacitance has an increasing trend as the volume duty cycle increases. This is due to the fact that the density of the foxtail millet increases when it is squeezed, with a consequent increase in the amount of foxtail millet per unit volume, and more electric field energy can be stored, so that the measuring instrument will measure a greater capacitance (Zhiheng et al., 2019; Wenchuan et al., 2023).

It can be seen from **Figure 6B** that at the same duty cycle (1/3), when the moisture content is constant, the value of the foxtail millet detection capacitance gets larger with the increase in temperature as a whole. The tendency to change is more pronounced in the high moisture content samples than in the low moisture content samples. The reason for this is that the rising temperature causes ionization of water molecules and the ion concentration goes up, which will accelerate the steering polarization of water molecules under the action of electric field. In the meanwhile, the rise in temperature enhances the thermal motion of water molecules, accelerating the orientation motion of polar molecules and the Brownian motion of free water within the foxtail millet. The generating polarization effect is greater than the thermal motion effect, which results in an increase in the relative dielectric constant of the foxtail millet, and therefore leads to an increase in its detection capacitance value.

### 3.3 Modeling and comparative analysis

In order to verify the reasonableness of the optimization algorithm, this study chooses six benchmark functions, i.e., F1, F2, F3, F4, F5, and F6, for testing, and sets the relevant parameters of the Logistic-SSA algorithm and the SSA algorithm to the same values. In this test, the population size is set to 30 and the number of iterations is set to 20 to compare and analyse the performance of the SSA algorithm before and after the improvement of the SSA algorithm using the Logistic algorithm.

The functional expressions for the six selected benchmark functions are given in Equations 22–27 below:


(22) $$f_{1}(x)=\sum_{i=1}^{n}x_{i}^{2}$$

(23) $$f_{2}(x)=\sum_{i=1}^{n}\left|x_{i}\right|+\prod_{i=1}^{n}\left|x_{i}\right|$$

(24) $$f_{3}(x)={\sum_{i=1}^{n}\left(\sum_{j=1}^{i}x_{j}\right)}^{2}$$

(25) $$f_{4}(x)=\max_{i}\left\{\left|x_{i}\right|,1\leq i\leq n\right\}$$

(26) $$f_{5}(x)=\sum_{i=1}^{n-1}\left[100{\left(x_{i+1}-x_{i}^{2}\right)}^{2}+{\left(x_{i}-1\right)}^{2}\right]$$

(27) $$f_{6}(x)={\sum_{i=1}^{n}\left(\left\lfloor x_{i}+0.5\right\rfloor \right)}^{2}$$

**Figure 7:**
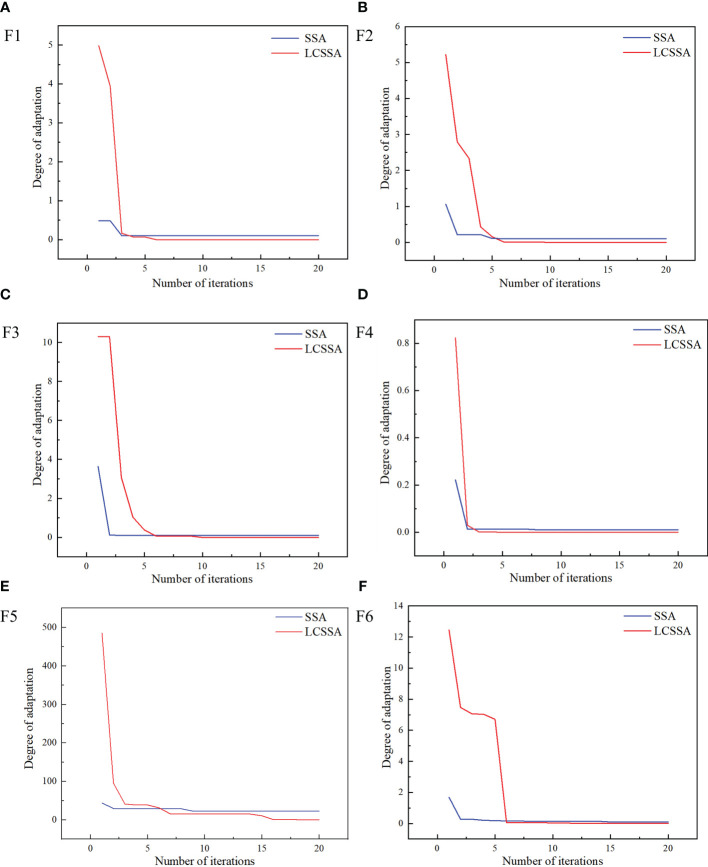
The iterative curves of SSA vs. LCSSA with 6 benchmark functions. Figure **(A)** shows the SSA and LCSSA iteration curves for the F1 function. Figure **(B)** shows the SSA and LCSSA iteration curves for the F2 function. Figure **(C)** shows the SSA and LCSSA iteration curves for the F3 function. Figure **(D)** shows the SSA and LCSSA iteration curves for the F4 function. Figure **(E)** shows the SSA and LCSSA iteration curves for the F5 function. Figure **(F)** shows the SSA and LCSSA iteration curves for the F6 function.

**Figure 7** below shows the iteration curves of the SSA algorithm and the Logistic-SSA (LCSSA) algorithm tested with the six benchmark functions presented above. It can be intuitively seen that the introduction of the optimization algorithm significantly improves the initial solution, improves the problem of SSA falling into local optimization, and at the same time reduces the minimum fitness value. Overall, the improved algorithm is closer to the ideal optimal solution, can better jump out of the local optimization, and effectively improves the performance of the optimization search.

In order to select the best detection model, the logistic-SSA algorithm and the SSA algorithm were used to optimize the BP and ELM algorithms, separately, for predicting the moisture content of the foxtail millets. The training set samples, and prediction set samples were randomly grouped in the ratio of 3:1. Using the empirical formula, as shown in Equation 28, to determine the number of nodes h in the hidden layer, the optimal prediction is searched for. In this case, the number of nodes in the input layer is set to 3 and the number of nodes in the output layer is set to 1.


(28) $$h=\sqrt{m+n}+a$$

Where, *m* is the number of nodes in the input layer, *n* is the number of nodes in the output layer and *a* is a constant between 1 and 10.

The temperature, volumetric duty cycle, and detection capacitance values were selected as independent variables for modelling and analysis, and the moisture content of the foxtail millet was chosen as the dependent variable to structure the model. The R, RMSE, RPD of the training set and prediction set for the foxtail millet moisture content estimation using Logistic-SSA-BP algorithm were 0.6404, 4.0130, 1.3020, and 0.5908, 4.8262, 1.2394, respectively. The R, RMSE, RPD of the training set and prediction set for the foxtail millet moisture content estimation using Logistic-SSA-ELM algorithm were 0.8022, 3.2887, 1.6751 and 0.7016, 3.7150, 1.4035, respectively. The prediction accuracy is significantly improved compared to both SSA-BP algorithm, BP algorithm and SSA-ELM algorithm, ELM algorithm. The use of differential capacitance sensors combined with deep learning algorithms can realize real-time and accurate detection of the foxtail millet moisture content, providing data support for precision agriculture, which is conducive to improving the quality and yield of foxtail millets. The results of the prediction accuracy of the established models are illustrated in **Table 2**, where it can be seen that both the Logistic-SSA-ELM algorithm and Logistic-SSA-BP algorithm have remarkably improved the prediction accuracy of the foxtail millet moisture content. The Logistic SSA-ELM algorithm has a good prediction effect on the moisture content of foxtail millet, which can be predicted to a certain extent.

**Table 2:** Modeling results based on different algorithms.

Models	Training set			Prediction set		
	R_C_	RMSE_C_	RPD_C_	R_P_	RMSE_P_	RPD_P_
BP	0.6035	4.1662	1.2541	0.5349	5.0542	1.1835
SSA-BP	0.6366	4.0295	1.2966	0.5835	4.8577	1.2314
Logistic-SSA-BP	0.6404	4.0130	1.3020	0.5908	4.8262	1.2394
ELM	0.7763	3.4727	1.5863	0.6826	3.8120	1.3683
SSA-ELM	0.7895	3.3810	1.6293	0.7009	3.7200	1.4021
Logistic-SSA-ELM	0.8022	3.2887	1.6751	0.7016	3.7150	1.4035

From the prediction results of different models in **Table 2**, the accuracy of the foxtail millet moisture content prediction model that was established using the Logistic-SSA-ELM algorithm was optimal. The predicted results of R_P_ were 0.0007 and 0.0190 higher than that of SSA-ELM and ELM respectively, reaching 0.8022. In comparison to SSA-ELM and ELM, RMSE_P_ were reduced by 0.0923 and 0.184 respectively, reaching 3.2887. As compared to SSA-ELM and ELM, RPD_C_ improved by 0.0458 and 0.0888 respectively, reaching 1.6751. Overall, the R_c_ and RPD_c_ values of the optimised model are improved over the original model and the RMSE_c_ value is reduced over the original model. The accuracy of the constructed model was high, and it could be used for rapid detection of moisture content in field foxtail millets. Using differentially structured capacitive sensors for moisture content measurement in foxtail millets is feasible and enables rapid on-line detection that can be extended to a wider range of applications.

**Figure 8:**
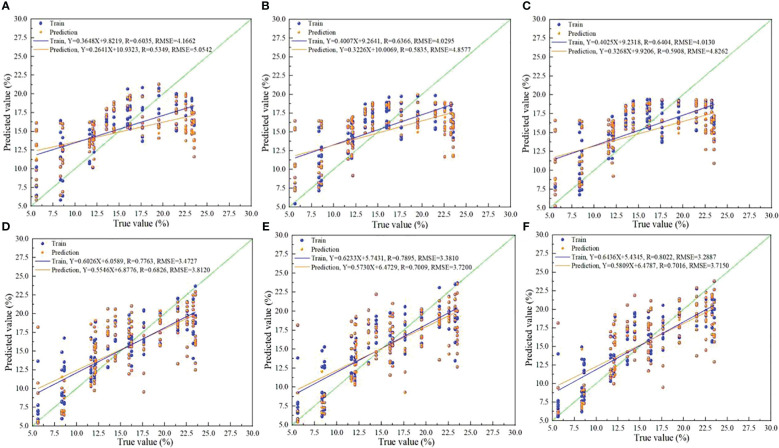
Plot of true vs. predicted values. Figure **(A)** shows a graph on the comparison of the true values and the predicted values output using the BP algorithm. Figure **(B)** shows a graph on the comparison of the true values and the predicted values output using the SSA-BP algorithm. Figure **(C)** shows a graph on the comparison of the true values and the predicted values output using the Logistic-SSA-BP algorithm. Figure **(D)** shows a graph on the comparison of the true values and the predicted values output using the ELM algorithm. Figure **(E)** shows a graph on the comparison of the true values and the predicted values output using SSA-ELM algorithm. Figure **(F)** shows a graph on the comparison of the true values and the predicted values output using Logistic-SSA-ELM algorithm.

The **Figure 8** exhibits the comparison diagram between the true values and the predicted values which are obtained by using different algorithms. Wherein, Figure (A) shows a graph on the comparison of the true values and the predicted values output using the BP algorithm. Figure (B) shows a graph on the comparison of the true values and the predicted values output using the SSA-BP algorithm. Figure (C) shows a graph on the comparison of the true values and the predicted values output using the Logistic-SSA-BP algorithm. Figure (D) shows a graph on the comparison of the true values and the predicted values output using the ELM algorithm. Figure (E) shows a graph on the comparison of the true values and the predicted values output using SSA-ELM algorithm. Figure (F) shows a graph on the comparison of the true values and the predicted values output using Logistic-SSA-ELM algorithm.

The above data processing and predictive modelling were done using Matlab software (USA, MathWorks) and plotted using Origin 2018 software.

## 4 Conclusion

In this paper, a differential capacitance sensor was designed in order to analyse foxtail millet under the influence of moisture content, ambient temperature, and volumetric duty cycle factors with the relationship to the measured capacitance value. Combining Logistic-SSA-BP and Logistic-SSA-ELM algorithms for model prediction of foxtail millet water content. The results indicate that the model predicted using the Logistic-SSA-ELM algorithm is more accurate. Meanwhile, it can also be seen that using differential capacitance sensors to detect the moisture content of grains is effective and has potential.

The pattern of change in the capacitance of foxtail millets at different moisture contents, ambient temperatures, and volume duty cycles was explored. In the moisture content range of 5.64% to 23.52%, the capacitance values increased with the increase in moisture content of the foxtail millets. In the temperature range of 15°C to 33°C, the foxtail millet capacitance increased with the increase of ambient temperature. In the volume duty cycle range of 0 to 1/2, the foxtail millet capacitance increased with the increase of volume duty cycle. The findings revealed that moisture content, ambient temperature and volumetric duty cycle have a notable effect on the capacitance values.

Logistic algorithm is introduced to optimize the Sparrow Search Algorithm (SSA), and then Back Propagation (BP) algorithm and Extreme Learning Machine (ELM) algorithm were optimized again respectively. The experimental results suggest that the ELM model optimized based on the Logistic-SSA algorithm is selected as the detection model of foxtail millet moisture content, and the predictive performance is satisfactory. The predicted results for R_C_, RMSE_C_, RPD_C_ and R_P_, RMSE_P_, RPD_P_ are 0.8022, 3.2887, 1.6751 and 0.7016, 3.7150, 1.4035 respectively. As seen, the predictive model has a high degree of accuracy. The method proposed in this paper can further improve the detection accuracy of the foxtail millet moisture content detection model, furthermore, this method provides thoughts and theoretical references for the prediction of moisture content of other crops. However, if the foxtail millet contains other conductive substances, such as metal particles, these substances may cause distortion of the capacitance measurement results. Therefore, care needs to be taken to avoid interference from conductive substances during the measurement. At the same time, capacitance measurement requires good electrode contact to ensure accurate measurement results. If the electrodes have poor contact or are loose, the measurement results may show large deviations.

## 5 Discussion

This paper presents a method for modelling differential capacitive sensors using Logistic-SSA-ELM algorithm. The method effectively reduces the influence of environmental disturbances on the measurement results and improves the measurement accuracy and reliability. Compared with other methods, capacitance sensors are highly sensitive, real-time, inexpensive, and capable of capturing small capacitance changes, providing a reliable means of accurately measuring foxtail millet moisture content, and providing strong support for improving agricultural production efficiency and resource utilisation. In addition, the study combines knowledge of electrical engineering and food science to provide a comprehensive study on the measurement and modelling of foxtail millet moisture content. An international audience may be interested in the novel algorithms and interdisciplinary applications of this study, as it has potential applications in food science and electrical engineering. However, this research has not yet been integrated with some mechanical devices such as combine harvesters for overall practical applications. Moreover, if the foxtail millet contains more impurities, dust, etc., it will interfere with the capacitance measurement results and may cause errors in the experimental results. These aspects need to be explored further to ultimately move towards modern agriculture. This research provides a viable approach to agricultural production that can help improve the efficiency and quality of foxtail millet production. Using the capacitance method, in combination with the designed capacitance sensor, provides an advanced and reliable solution for the measurement of moisture content in foxtail millet. This technology has great potential to promote modernisation of agriculture, increase the efficiency of agricultural production and enable smart agriculture. Through enabling precision measurements, it provides a new way to manage the quality and optimise the yield of foxtail millets, laying the foundation for sustainable agriculture and precision farming.

## Data availability statement

The original contributions presented in the study are included in the article/supplementary material. Further inquiries can be directed to the corresponding authors.

## Author contributions

ZQ: Conceptualization, Data curation, Methodology, Software, Validation, Visualization, Writing – original draft, Writing – review & editing, Formal analysis, Investigation, Project administration. GL: Supervision, Writing – review & editing. ZH: Supervision, Writing – review & editing. XH: Supervision, Writing – review & editing. ZZ: Writing – review & editing. ZL: Funding acquisition, Writing – review & editing. HD: Resources, Writing – review & editing.
